# Genome editing in rice

**DOI:** 10.1186/s12284-020-00384-6

**Published:** 2020-05-14

**Authors:** Masaki Endo, Seiichi Toki

**Affiliations:** Institute of Agrobiological Sciences, National Agriculture and Food Research Organization, Tsukuba, Japan

Rice is a major staple food that sustains more than three billion people in the world. However, as the world’s population continues to grow, it has become more and more urgent to develop super high yielding varieties as well as hyper-tolerant varieties to pathogens and climate change. Genome editing technology is gradually revolutionizing crop improvement by facilitating a rapid, efficient and simple strategy for modification of target genes. Since many genes and single nucleotide polymorphisms (SNPs) involved in agronomically important traits have already been determined by comparative genomics, GWAS and OMICS-based approaches, genome editing could provide the ultimate tool to accelerate the breeding of new mutations that has until now been conducted by random mutagenesis.

There are two types of genome editing technology, namely, targeted mutagenesis and gene targeting. In targeted mutagenesis, DNA double-strand breaks (DSBs) are induced at targeted sequence(s) using site specific nucleases (SSNs) such as CRISPR/Cas9. The targeted mutations can then be generated by the error-prone non-homologous end joining pathway resulting mainly on insertions and deletions. However, recent advances in the development of base editors have allowed specific induction of individual base substitutions. Gene targeting, on the other hand, uses the homologous recombination pathway, in which DSBs induced spontaneously or specifically by SSNs can be repaired using exogenously supplied donor DNA. Thus, modification of a targeted gene, including specific substitutions, insertions and deletions of desired sequences can be conducted more precisely.

Rice is one of the most preferred plants for genome editing due to its small genome size, as well as the availability of genome sequences and cell and genome engineering techniques, including an efficient protoplast culture and transformation systems. A universal plant gene targeting system using positive/negative selection has been developed exclusively in rice (Terada et al. 2002; Osakabe et al. 2014; Nishizawa-Yokoi et al. 2015a). Furthermore, marker-free gene-targeted rice harboring only the desired mutation in the targeted locus can be generated by precise excision of the positive selection marker gene using the *piggyBac* transposon system (Nishizawa-Yokoi et al. 2015b). In addition, the CRISPR/Cas system was pioneered in rice (Shan et al. 2013), and recent improvements to this system were also applied first in rice (Shimatani et al. 2017; Zong et al. 2017; Li et al. 2018; Endo et al. 2019).

In this special issue, we have compiled some of the recent advances in using genome editing strategy for rice improvement. The technical papers focused on the most commonly used CRISPR/Cas system, Cas9 which creates blunt-end DNA breaks, and Cas12a which creates sticky ends suitable for molecular surgery. Banakar et al. succeeded in targeted mutagenesis by biolistic delivery of Cas9 or Cas12a ribonucleoprotein (RNP) into mature seeds derived rice embryos. Zhong et al. tried to generate guide RNA (gRNA) from an intron sequence, and succeeded in targeted mutagenesis by expressing both Cas protein (Cas9 or Cas12a) and gRNA sequence as a single transcriptional unit. In this system, the Cas protein and gRNA should be co-expressed in the same cell.

Although targeted mutagenesis has been used mainly to produce loss-of-function mutants, two groups have successfully generated gain-of-function mutants by CRISPR/Cas9-mediated mutagenesis. Endo et al. succeeded in beta-carotene fortification of rice calli by directed gene modification of the intron/exon junction sequence of the rice orange gene (OsOr). This report suggested that beta-carotene can be accumulated via a non-transgenic approach. Akama et al. succeeded in increasing the gamma-aminobutyric acid content in rice grains by targeted deletion of the calmodulin-binding domain (CaMBD) from the rice glutamate decarboxylase 3 (OsGAD3) gene. In this case, the CaMBD coding sequence could be trimmed off using a pair of gRNAs.

Also in this issue, Yu et al. reviewed some of the new techniques for genome editing and the identification of marker-free genome-edited mutants in monocot crops. Also, Vu et al. suggested potential approaches for the improvement of gene targeting in monocot crops, and discussed regulation of genome-edited products.

We hope that the topics covered in this special issue will enhance the use of genome editing technology, not only for understanding plant systems but also for improvement of rice yield and quality.

Masaki Endo and Seiichi Toki.

Guest Editors.

## References

[CR1] Endo M, Mikami M, Endo A, Kaya H, Itoh T, Nishimasu H, Nureki O, Toki S (2019). Genome editing in plants by engineered CRISPR-Cas9 recognizing NG PAM. Nat Plants.

[CR2] Li C, Zong Y, Wang Y, Jin S, Zhang D, Song Q, Zhang R, Gao C (2018). Expanded base editing in rice and wheat using a Cas9-adenosine deaminase fusion. Genome Biol.

[CR3] Nishizawa-Yokoi A, Endo M, Ohtsuki N, Saika H, Toki S (2015). Precision genome editing in plants via gene targeting and piggyBac-mediated marker excision. Plant J.

[CR4] Nishizawa-Yokoi A, Nonaka S, Osakabe K, Saika H, Toki S (2015). A universal positive-negative selection system for gene targeting in plants combining an antibiotic resistance gene and its antisense RNA. Plant Physiol.

[CR5] Osakabe K, Nishizawa-Yokoi A, Ohtsuki N, Osakabe Y, Toki S (2014). A mutated cytosine deaminase gene, codA (D314A), as an efficient negative selection marker for gene targeting in rice. Plant Cell Physiol.

[CR6] Shan Q, Wang Y, Li J, Zhang Y, Chen K, Liang Z, Zhang K, Liu J, Xi JJ, Qiu JL, Gao C (2013). Targeted genome modification of crop plants using a CRISPR-Cas system. Nat Biotechnol.

[CR7] Shimatani Z, Kashojiya S, Takayama M, Terada R, Arazoe T, Ishii H, Teramura H, Yamamoto T, Komatsu H, Miura K, Ezura H, Nishida K, Ariizumi T, Kondo A (2017). Targeted base editing in rice and tomato using a CRISPR-Cas9 cytidine deaminase fusion. Nat Biotechnol.

[CR8] Terada R, Urawa H, Inagaki Y, Tsugane K, Iida S (2002). Efficient gene targeting by homologous recombination in rice. Nat Biotechnol.

[CR9] Zong Y, Wang Y, Li C, Zhang R, Chen K, Ran Y, Qiu JL, Wang D, Gao C (2017). Precise base editing in rice, wheat and maize with a Cas9-cytidine deaminase fusion. Nat Biotechnol.

